# *Bacillus Coagulans *GBI-30 (BC30) improves indices of *Clostridium difficile*-Induced colitis in mice

**DOI:** 10.1186/1757-4749-3-16

**Published:** 2011-10-20

**Authors:** Leo R Fitzpatrick, Jeffrey S Small, Wallace H Greene, Kelly D Karpa, David Keller

**Affiliations:** 1 Department of Pharmacology, Penn State College of Medicine, 1214 Research Boulevard, Hummelstown, PA 17036, USA; 2 Department of Pathology, Penn State College of Medicine, PO Box 850, Hershey, PA 17033, USA; 3 Ganeden Biotech Inc., 5915 Landerbrook Drive, Suite 304, Mayfield Heights OH 44124, USA

**Keywords:** *Clostridium difficile*, probiotics, colitis, mice

## Abstract

**Background:**

Probiotics have beneficial effects in rodent models of *Clostridium difficile *(*C. diffiicle*)-induced colitis. The spore forming probiotic strain *Bacillus Coagulans *GBI-30, 6086 (BC30) has demonstrated anti-inflammatory and immune-modulating effects *in vitro*. Our goal was to determine if BC30 improved *C. difficile*-induced colitis in mice. Starting on study day 0, female C57BL/6 mice were dosed by oro-gastric gavage for 15 days with vehicle (saline) or BC30 (2 × 10^9 ^CFU per day). Mice in the *C. difficile *groups received an antibiotic mixture (study days 5 to 8 in the drinking water), and clindamycin (10 mg/kg, i.p., on study day 10). The *C. difficile *strain VPI 10463 was given by gavage at 10^4 ^CFU to induce colitis on day 11. On day 16, stools and colons were collected for further analyses.

**Results:**

All mice treated with BC30 survived on study day 13, while two mice treated with vehicle did not survive. On day 12, a significant difference (p = 0.0002) in the percentage of mice with normal stools (66.7%) was found in the BC30/*C. difficile *group, as compared to the vehicle/*C. diffcile *group (13.0%). On study day 16, 23.8% of mice treated with BC30 had normal stools, while this value was 0% with vehicle treatment (p value = 0.0187). On this day, the stool consistency score for the BC30/*C. difficile *group (1.1 ± 0.2) was significantly lower (p < 0.05) than for the vehicle/*C. difficile *cohort (1.9 ± 0.2). BC30 modestly attenuated the colonic pathology (crypt damage, edema, leukocyte influx) that was present following *C. difficile infection*. Colonic MIP-2 chemokine contents (pg/2 cm colon) were: 10.2 ± 0.5 (vehicle/no *C. difficile*), 24.6 ± 9.5 (vehicle/*C. difficile*) and 16.3 ± 4.3 (BC30/*C. difficle*).

**Conclusion:**

The probiotic BC30 improved some parameters of *C. difficile*-induced colitis in mice. BC30 prolonged the survival of *C. diffiicle *infected mice. Particularly, this probiotic improved the stool consistency of mice, in this infectious colitis model.

## Background

*Clostridium Difficile *(*C. difficile*) infection can cause nosocomial-related diarrhea [1]. The spectrum of *C. difficile*-associated disease (CDAD) ranges from mild antibiotic associated diarrhea to severe (or even life threatening) pseudomembranous colitis [1]. CDAD is caused by the actions of two exotoxins (toxin A and toxin B), which are produced by pathogenic strains of *C. difficile *[2,3].

Previous data suggests that toxin A can activate the nuclear factor-kappa B (NF-κB) signal transduction system in monocytes and colonic epithelial cells [4,5]. This activation of NF-κB leads to secretion of a key pro-inflammatory chemokine (IL-8) and subsequently to neutrophil influx into the colonic tissue [4,5]. Neutrophils play a key role in the pathogenesis of CDAD, both in humans and in mice [6].

CDAD is often treated successfully with standard antibiotics such as vancomycin, or metronidazole [7,8]. However, recurrence occurs in many patients [6,8]. Some clinical studies have focused on combined treatment with vancomycin and probiotics such as *Saccharomyces boulardii *for recurrent disease [8-11]. Therefore, initial treatment regimens with probiotics, or their use for prevention of recurrent disease, may be attractive as part of the overall therapeutic strategy for CDAD [8-11].

Probiotics are live microorganisms which, when ingested, can confer health benefits [12]. Typically, probiotics include various strains of *Lactobacillus *and/or *Bifidobacteria *species. They exist as either single entities or as combination products (e.g., VSL #3) [13,14]. Other known probiotics include certain non-pathogenic *Escherichia coli *(*E. coli*) strains like Nissle 1917 and M-17 [13,15]. Mechanisms explaining the potential role of probiotics as anti-colitis therapies have been reviewed in detail elsewhere [13]. Recently, our laboratory has shown that a non-pathogenic strain of *E. coli *can inhibit colitis in mice by immunomodulating the NF-κB signal transduction system and inhibiting associated pro-inflammatory cytokines [15]. Of note, a recent paper found that *Lactobacillus acidophilus *was effective for treating CDAD in mice [16].

Of direct relevance to this study, the novel spore forming probiotic strain GanedenBC^30 ^(*Bacillus coagulans *GBI-30, 6086) is relatively resistant to extreme temperatures, as well as stomach acidity, digestive enzymes and bile salts [17]. *Bacillus Coagulans GBI-30 *(BC30) has been used for human consumption to ameliorate symptoms in various gastrointestinal disorders, as well as an immunomodulating agent in *ex-vivo *viral challenge, and *in vivo *human immunodeficiency virus research [17,18].

Preliminary research to articulate its mechanisms of action demonstrated anti-inflammatory and immunomodulating effects *in vitro *[19]. Therefore, as a logical extension to this *in vitro *evaluation, we evaluated the effectiveness of BC30 for inhibiting *C. difficile *induced colitis in mice. Since NF-κB activation and chemokine secretion also play important roles in the pathogenesis of CDAD, we also evaluated the effects of BC30 on this critical transcription factor, as well as MIP-2, in this murine CDAD model [4-7].

## Results

### BC30 prolongs mouse survival after the administration of *C. difficile*

**Figure 1:**
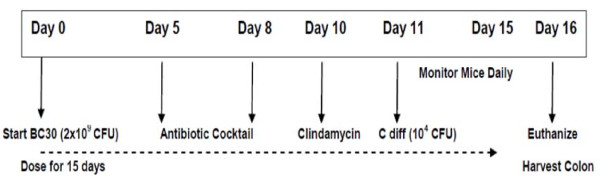
**Study Overview**. This figure shows an overview of the key events associated with the *Clostridium diff*icile induced colitis model that was used for this study. Female C57BL/6 mice were dosed during study days 0 through 15 with 2 × 10^9 ^CFU of BC30, or vehicle (0.9% saline). On study days 5 through 8, mice were given an antibiotic coktail in the drinking water as described in the Methods section. On day 10, clindamycin was administered i.p. at a dose of 10 mg/kg. On day 11, mice received either *Clostridium diff*icile (designated as C diff) [1 × 10^4 ^CFU of VPI 10463], or vehicle, by oro-gastric gavage. Body weight and stool consistency data were collected daily on study days 11 through 16. On study day 16, mice were euthanized and the colons were removed for measuring morphometric, biochemical and histological indices of colitis.

**Figure 2:**
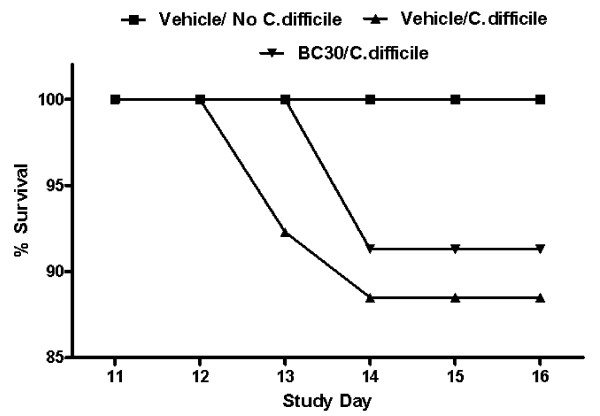
**Mouse survival data**. All mice (6/6) that did not receive *C. difficile *survived for the duration of the study. As shown, 100% of mice treated with BC30 survived on study day 13, while 2 mice treated with vehicle did not survive on that day (92.3% survival). By day 14, 21/23 mice survived in the BC30/C. *difficile *treatment group, while 23/26 mice survived in the vehicle/*C. difficile *treatment group. Therefore, the cumulative survival rates in the study were: 100% (Vehicle/no *C. difficile*), 88.5% (Vehicle/*C. difficile*) and 91.3% (BC30/*C. difficile*). However, there was not a statistically significant difference in the survival rate between any of the treatment groups.

Figure 1 shows an overview of the key events associated with the *C. diff*icile induced colitis model that was used for this study. As shown in Figure 2, all mice treated with BC30 (100%) survived on study day 13, while 2 mice treated with vehicle did not survive on that day (92.3% survival). By day 14, 21/23 mice survived in the BC30/C. *difficile *treatment group, while 23/26 mice survived in the Vehicle/*C. difficile *treatment group. As expected, all mice (6/6) that did not receive *C. difficile *survived for the duration of the study. Therefore, the cumulative survival rates in the study were: 100% (Vehicle/no *C. difficile*), 88.5% (Vehicle/*C. difficile*) and 91.3% (BC30/*C. difficile*). However, there was not a statistically significant difference in the survival rate between any of the treatment groups.

Despite the delay in mortality rate in the BC30 treatment group, there were similar body weight profiles in both *C. difficile *treatment groups. Mice in these treatment groups lost weight (by approximately 10%) between days 11 and 14, and then subsequently began to gain weight. There were no differences in the body weights (grams) on day 16: 19.5 ± 0.4 (Vehicle/*C. difficile*) and 18.8 ± 0.4 (BC30/*C. difficile*). In contrast, mice that did not receive *C. difficile *gained weight during the study period (body weight on day 16 = 20.6 ± 0.5 grams).

### BC30 treatment significantly improves the stool consistency in *C. difficile* infected mice

**Figure 3:**
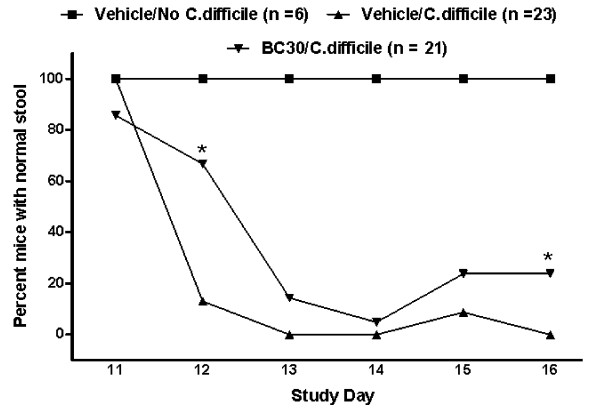
**Stool consistency data**. All mice that did not receive C. difficile had normal stools throughout the study. In contrast, only 13% of mice in the *Vehicle/C. difficile *treatment group had normal stools on day 12, while 67% of the BC30 treated mice still had normal stools. On days 13 and 14, altered stool consistency was seen in both *C. difficile *treatment groups. However, evidence of a normal stool was seen in 24% of BC30 treated mice on day 16, but no vehicle treated animals (0%) showed a normal stool consistency on that day. Statistical significance (* p < 0.05) between the vehicle and BC30 treatment groups was found on study days 12 and 16.

Figure 3 illustrates that 13% of mice in the *Vehicle/C. difficile *treatment group had normal stools on day 12; while 67% of the probiotic (BC30) treated mice still had normal stools. On days 13 and 14, altered stool consistency was seen in both *C. difficile *treatment groups. However, evidence of a normal stool was seen in 24% of BC30 treated mice on day 16, but no vehicle treated animals (0%) showed a normal stool consistency on that day. Statistical significance (p < 0.05) between the vehicle and BC30 treatment groups was found on study days 12 and 16.

**Figure 4:**
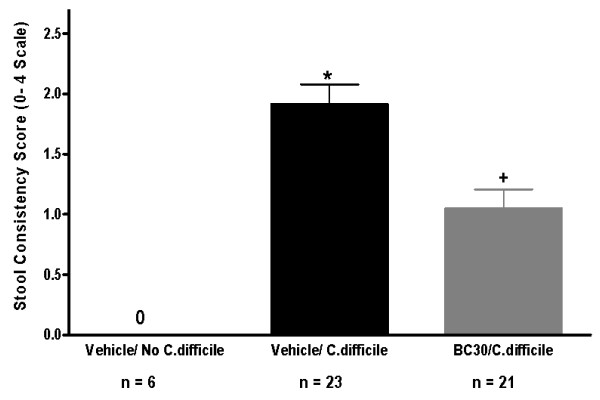
**Stool consistency score data**. The mean stool consistency scores for the three cohorts of mice on day 16 were: 0 ± 0 (Vehicle/No *C. difficile*), 1.9 ± 0.2 (Vehicle/*C. difficile*) and 1.1 ± 0.2 (BC30/*C. difficile*). * indicates p < 0.05 vs. Vehicle/no *C. difficile*. + indicates p < 0.05 vs. Vehicle/*C. difficile*.

Moreover, as shown in Figure 4, the mean stool consistency score (day 16) was significantly lower in BC30/*C. difficile *treated mice (1.1 ± 0.2) than in corresponding Vehicle/*C. difficile *treated animals (1.9 ± 0.2).

### BC30 slightly attenuates indices of *C. difficile*-induced colonic pathology

The distal colonic weight was significantly increased in mice treated with vehicle plus *C. difficile*. However, treatment with the probiotic (BC30) only slightly normalized the colonic weight. Values (mg/cm colon) on day 16 were: 17.4 ± 0.8 (Vehicle/no *C. difficile*), 26.3 ± 0.7 (Vehicle/*C. difficile*) and 25.4 ± 0.6 (BC30/*C. difficile*). A statistically significant difference (p < 0.05) was found between the Vehicle/no *C. difficile and *Vehicle/*C. difficile *treatment groups, but not between the two *C. difficile *treated groups of mice.

**Figure 5:**
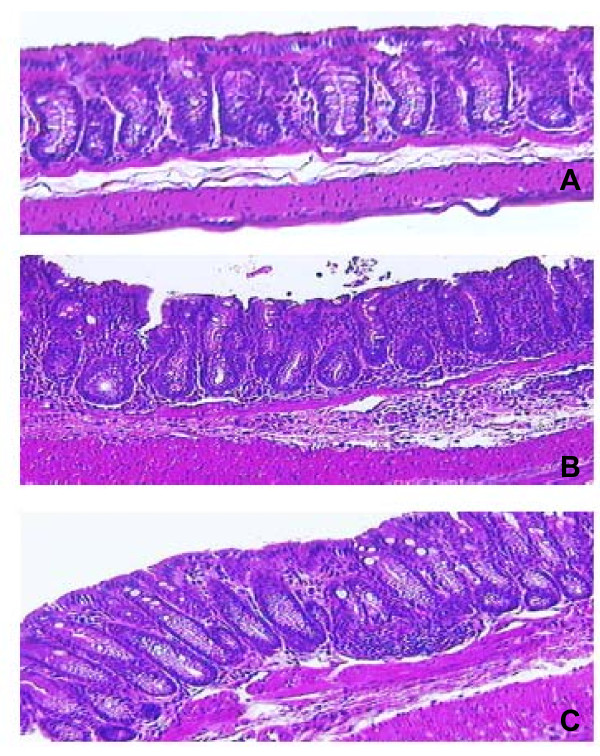
**Representative colonic histology pictures (day 16)**. The pictures are from hematoxylin and eosin (H&E) stained colonic specimens, at a magnification of 200-fold. Panel A shows a normal histological appearance within the colon of a mouse that was not infected with *C. difficile*. Panel B is from the colon of a Vehicle/*C. difficile *treated mouse. There is evidence of crypt damage, submucosal edema and the influx of inflammatory cells in the lamina propria and sub-mucosa. Panel C is from the colon of a BC30/*C. difficile *treated animal. Although, colonic pathology is still present, it is less prominent than in panel B.

Representative histology pictures are shown in Figure 5. *C. difficile *infection caused altered colonic histopathology. Specifically, crypt damage, submucosal edema and the influx of inflammatory cells in the lamina propria and sub-mucosa were evident in these mice (panel B). Overall, BC30 treatment resulted in a modest attenuation of the colonic histological pathology observed in Vehicle/*C. difficile *treated mice (panel C). The overall colonic histology scores on study day 16 were: 2.55 ± 0.39 (Vehicle/No *C. difficile*), 5.19 ± 0.22 (Vehicle/*C. difificile*) and 4.96 ± 0.34 (BC30/*C. difficile*). A statistically significant difference (p < 0.05) was found between the Vehicle/no *C. difficile and *Vehicle/*C. difficile *treatment groups, but not between the two *C. difficile *treated groups of mice.

### BC30 attenuates colonic NF-kB p65 binding and chemokine content in *C. diffiicle *infected mice

**Figure 6:**
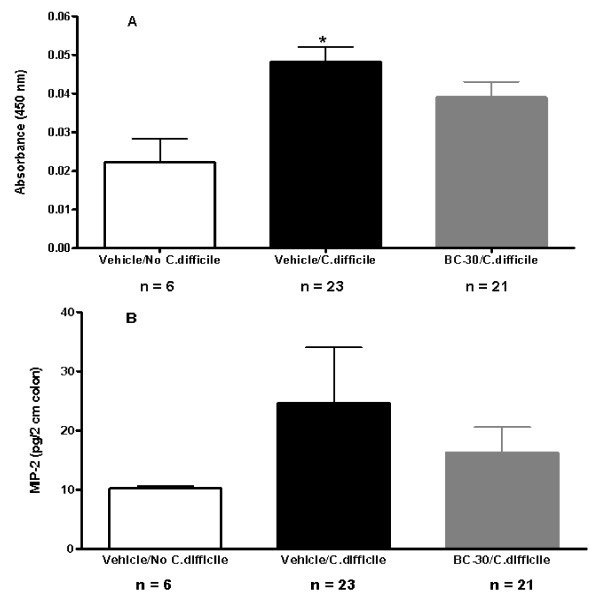
**Panel A shows the colonic NF-κB p65 data**. The mean absorbance values for p65 binding were: 0.022 ± 0.006 (Vehicle/no *C. difficile*), 0.048 ± 0.004 (Vehicle/*C. difficile*) and 0.039 ± 0.004 (BC30/*C. difficile)*. The * symbol indicates p < 0.05 vs. Vehicle/no *C. difficile*. **Panel B **shows the colonic MIP-2 chemokine data. The MIP-2 values (pg/2 cm colon) were: 10.2 ± 0.5 (Vehicle/no *C. difficile*), 24.6 ± 9.5 (Vehicle/*C. difficile*) and 16.3 ± 4.3 (BC30/*C. difficile)*.

As shown in Figure 6, there were reductions in the colonic nuclear binding of p65 and the colonic MIP-2 content respectively, when mice were treated with BC30. The mean absorbance values for p65 binding were: 0.022 ± 0.006 (Vehicle/no *C. difficile*), 0.048 ± 0.004 (Vehicle/*C. difficile*) and 0.039 ± 0.004 (BC30/*C. difficile)*. Specifically, as shown in Figure 6A, the nuclear binding of NF-κB p65 was increased 2.2 fold in the colons of Vehicle/*C. difficile *treated mice, but only 1.8 fold in probiotic treated animals. Similarly, the colonic MIP-2 content was significantly increased in vehicle treated mice, but the increase was less dramatic in BC30 treated mice (Figure 6B). On study day 16, the MIP-2 values (pg/2 cm colon) were: 10.2 ± 0.5 (Vehicle/no *C. difficile*), 24.6 ± 9.5 (Vehicle/*C. difficile*) and 16.3 ± 4.3 (BC30/*C. difficile)*. Due to the variability in the colonic MIP-2 values within the Vehicle/*C. difficile *treatment group, a statistically significant reduction in the colonic MIP-2 content was not found with BC30 treatment.

## Discussion

Recently, Chen et al. described a murine model of CDAD that could be used for testing the efficacy of applicable pharmacological agents (antibiotics, probiotics) [6]. Infection of female C57BL/6 mice with 10^3 ^to 10^4 ^CFU of *C. dififcile *was associated with significant weight loss, diarrhea and mortality [6]. In a similar fashion, we also showed that the infection of vehicle treated mice resulted in transient weight loss, altered stool consistency and some evidence of mortality (11.5%).

In contrast to vehicle treatment, mice treated with the probiotic (BC30) had a delayed onset in mortality (no deaths until day 14), as well as a slightly reduced overall mortality rate (8.7%), when compared to vehicle treated animals (Figure 2). In a similar fashion, mice treated with BC30 had a delayed onset in the appearance of altered stool consistency (Figure 3). Specifically, on day 12, 87% of vehicle treated mice had evidence of loose stools or diarrhea. In contrast, only 33% of BC30 treated animals had evidence of altered stool consistency (p < 0.05 vs. vehicle). Moreover, on the final study day (day 16), BC30 treated mice still had a higher incidence of normal stools (Figure 3), as well as a significantly lower stool consistency score (Figure 4). These results demonstrate evidence of improved stool consistency in *C. difficile *infected mice that were pre-treated with BC30.

Murine CDAD is associated with a specific colonic histopathology that includes crypt damage, submucosal edema and the influx of inflammatory cells [6]. These pathological changes were also evident in our Vehicle/*C. diificle *treated cohort of mice (panel B, Figure 5). In contrast, mice treated with BC30 showed some evidence of attenuated colonic histopathology, including decreased leukocyte influx into the colon (panel C, Figure 5). However, the overall comparisons of mean colonic histology scores were not statistically different on day 16.

Data from other studies suggests that toxin A secreted by *C. difficile *can activate the NF-κB signal transduction system in monocytes and colonic epithelial cells [4,5]. This activation of NF-κB leads to the secretion of a key pro-inflammatory chemokine (IL-8) and subsequently neutrophil influx into the colonic tissue [4,5]. Interestingly, BC-30 can significantly inhibit the IL-8 directed migration of human neutrophils *in vitro *[19]. Based on these results, we measured the effects of BC30 on the nuclear binding of NF-κB p65, as well the murine chemokine (MIP-2) content in the colons of *C. difficile *infected mice. Probiotic treatment resulted in reductions of both colonic p65, as well as the MIP-2 content (Figure 6). However, statistical significance was not achieved compared to values in vehicle treated mice.

Nevertheless, these effects of BC30 on NF-κB mediated pathological processes (Figure 6) may contribute to the observed improvement in stool consistency observed in the probiotic-treated mice. For example, NF-κB activation is involved in the up-regulation of Fas-ligand, which subsequently leads to colonocyte apoptosis [20]. Colonocyte apoptosis could diminish the barrier function of the colonic mucosa, and contribute to the altered stool consistency associated with CDAD [20]. By reducing the colonic activation of NF-κB (Figure 6), it is possible that BC-30 treatment improved the barrier function of the colonic mucosa (Figure 5C), thereby improving stool consistency (Figures 2 and 3). Possibly, the improvement in stool consistency was unrelated to direct effects on colonization of *C. difficile*, or an alteration in the production of toxins A and B, because all infected mice showed evidence of infection and exotoxin production by ELISA (data not shown). Nevertheless, the ELISA kit utilized in this study does not quantify either the numbers of *C. difficile *in the colon, or the actual amounts of toxin production. Therefore, it is also possible that BC30 attenuated the level of *C. difficile *colonization and/or production of toxins in the colon. Future studies are needed to better understand the mechanisms, by which BC30 favorably impact stool consistency, as we observed in this study.

Of importance to this study, it is probable that the use of antibiotics in this murine CDAD model (during study days 5 to 10) resulted in anti-microbial effects that altered the levels of BC30 in the colon (David Keller, personal communication). Therefore, future studies with this murine model of CDAD should focus on effects of BC30 on the recurrence of *C. difficile *following treatment with vancomycin [6]. Using this recurrence paradigm, the unwanted anti-microbial effects of antibiotics will not negatively impact the presence of BC-30 in the mouse colon. Finally, it would also be interesting to test other *Bacillus coagulans *strains in this type of experimental paradigm.

## Conclusions

The probiotic BC30 improved some parameters of *C. difficile*-induced colitis in mice. BC30 prolonged the survival of *C. diffiicle *infected mice. Particularly, this probiotic improved the stool consistency of mice, in this infectious colitis model. Our results support the concept that probiotics like BC30 may find a niche for the treatment of CDAD.

## Methods

### *Bacillus Coagulans *GBI-30, 6086 (BC30)

BC30 was obtained from Ganeden Biotech Inc. (Mayfield Heights, OH).

### Murine *Clostridium difficile*-Induced Colitis

We followed the protocol developed by Chen et al., with slight modifications [6]. BC30 (2 × 10^9 ^CFU per day), or vehicle (0.9% saline), was dosed by oro-gastric gavage from study day 0 until study day 15. Both body weight and stool consistency data were collected daily on study days 11 through 16. Stool samples from all mice were scored based on the consistency of the fecal sample, as shown here: 0 = normal, 1 = loose stool, 2 = loose/some diarrhea, 3 = diarrhea and 4 = severe watery diarrhea.

On day 16, we confirmed the presence of *Clostridium difficile *and associated toxins (A and B) with a Wampole™ CD quick check complete kit from Inverness Medical (Princeton, NJ). On this study day, mice were euthanized; and the distal colon was collected for evaluating morphometric (colon weight), histological and biochemical parameters. An overview of the study design is shown in Figure 1. This protocol was approved by the Internal Animal Care and Use Committee (IACUC) at the Penn State College of Medicine.

### Colonic Histology Evaluation

Using coded slides from the distal colon, four areas from each slide were scored on a three-point severity scale: 0 = Normal, 1 = Mild, 2 = Moderate, 3 = Severe, for three different parameters. These three parameters were epithelial damage, mucosal/submucosal edema and leukocyte infiltration. Therefore, the total score for each slide (i.e., mouse) was between 0 and 9.

### Colonic NF-κB p65 Assay

We utilized a TransAM™ NF-κB p65 assay kit from Active Motif (Carlsbad, CA). This assay measures the nuclear binding of p65 to a consensus NF-κB binding site. For the assay, we used 20 μg of protein from colonic nuclear extracts. The results are expressed as the absorbance at 450 nm, as described previously by our laboratory [15].

### Colonic MIP-2 Content

MIP-2 (macrophage inflammatory protein-2) is a functional murine homolog of the human chemokine, IL-8. The colonic MIP-2 content was measured with an ELISA kit from R&D systems (Minneapolis, MN). Results are expressed as pg/2 cm colon.

### Statistical Analyses

Statistical analyses were done with a GraphPad Prism^® ^software program (San Diego, CA). All data are expressed as the mean ± SEM. Differences in mouse survival, as well as the percentages of mice with normal stools, were determined with the Fisher's exact test. Stool consistency scores and colonic histology scores were evaluated with the Kruskal-Wallace test, followed by Dunn's test to compare individual treatment groups. All other parameters were evaluated by ANOVA, followed by the Newman Keuls test for individual treatment groups. A p value of < 0.05 was considered to be statistically significant for all parameters.

## List of Abbreviations

BC30: *Bacillus Coagulans GBI-30*; NF-κB: Nuclear Factor-kappa B; CDAD: *Clostridium difficile*-associated disease; MIP-2: macrophage inflammatory protein-2.

## Competing interests

None of the authors have any conflict of interest disclosures to make regarding this manuscript, with the exception of Dr. David Keller. Dr. Keller is a paid employee of Ganeden Biotech Inc.

## Authors' contributions

LRF contributed to the technical and intellectual aspects of the manuscript. WHG, KDK and DK contributed to the intellectual aspects of the paper. JSS contributed to the technical aspect of the manuscript. All the authors read and approved the manuscript.
